# *Vitess 3.8*: a modernized framework for Monte Carlo neutron tracing simulations

**DOI:** 10.1107/S1600576726001093

**Published:** 2026-03-28

**Authors:** José Ignacio Robledo, Nicoló Violini, Fabian Beule, Jorg Voigt, Paul Zakalek, Klaus Lieutenant

**Affiliations:** a Jülich Centre for Neutron Science, Forschungszentrum Jülich GmbH, Wilhelm-Johnen Strasse, 52425Jülich, Germany; b Department of Physics (CAB) National Scientific and Technical Research Council (CONICET) 8400 Bariloche Argentina; Technical University of Denmark, Denmark

**Keywords:** Monte Carlo simulation, neutron ray tracing, neutron scattering, computer programs

## Abstract

*VITESS* is a modular Monte Carlo simulation package for modeling and optimizing neutron scattering instruments through ray tracing of neutrons. The latest release, *VITESS 3.8*, introduces major advances in functionality and software infrastructure. They include new source modules for integrating external moderator simulations and artificial-intelligence-based flux modeling, as well as prism for beam deflection and crystalline material simulations via *NCrystal*. Existing modules have been extended to support spin-dependent scattering, advanced monochromator motions and improved polarization handling. The update also includes a Git-based continuous integration system ensuring reproducibility.

## Abstract 2

*VITESS 3.8* introduces modernized neutron-source modeling with new artificial-intelligence-based and *KDSource* modules, plus expanded component support including a prism module and *NCrystal*-integrated sample module. It also delivers major upgrades to existing modules and multiple new features, advancing the simulation framework.

## 1. Introduction

*VITESS* (virtual instrumentation tool for the ESS) is a modular Monte Carlo simulation package designed for the modeling, optimization and performance analysis of neutron scattering instruments and virtual experiments (Zsigmond *et al.*, 2002 ▸; Zendler *et al.*, 2014 ▸; https://vitess.fz-juelich.de). Developed originally to support the design of instruments for the European Spallation Source (ESS), *VITESS* has evolved into a versatile and widely used framework for simulating neutron propagation through complex instrument geometries.

The software implements a ray-tracing approach, in which neutrons (particles characterized by their position, direction, wavelength and polarization) are described by rays. Each ray corresponds to a discrete number of physical neutrons. These virtual neutron rays are propagated through a sequence of instrument components—such as guides, choppers, monochromators and detectors—each represented by a dedicated simulation module, with which they are allowed to interact. Every neutron ray carries a weight (or probability) that can be decreased after each interaction, describing how many neutrons are still left in the instrument at a given point. *VITESS*’s modular structure allows researchers to flexibly assemble and modify virtual instruments, facilitating systematic studies of instrument performance and design trade-offs as well as virtual experiments.

One of the key strengths of *VITESS* lies in its balance between physical accuracy and computational efficiency. The tool accounts for the essential physics of neutron interactions with instrument components while remaining sufficiently fast for extensive parameter studies. It supports both continuous sources and pulsed source time structures, enabling the realistic simulation of modern neutron facilities. The software’s ability to interface with data formats such as Monte Carlo Particle Lists (MCPL) (Kittelmann *et al.*, 2017 ▸) further enhances interoperability with other simulation tools and data analysis frameworks.

Over the years, *VITESS* has contributed significantly to the conceptual and technical development of neutron scattering instruments at major facilities worldwide. Its open and extensible design makes it particularly valuable for both early-stage instrument design and virtual experiments aimed at testing new concepts in neutron optics and detector technologies.

The release of *VITESS 3.8* marks a significant update to the long-standing Monte Carlo package, integrating new capabilities aimed at improving both physical modeling and software usability. This version introduces four new modules, notably two source components—kd_source and source_AI (Section 3.5)—that enhance interoperability with external target–moderator simulations such as those performed with *MCNP* (Forster & Godfrey, 2006 ▸) by leveraging artificial intelligence (AI) models. Additional modules include prism, which simulates refraction on arrays of triangular prisms for beam deflection (Section 3.8), and sample_ncrystal, providing direct access to the *NCrystal* library (Cai & Kittelmann, 2020 ▸) for realistic crystalline material modeling (Section 3.2). Several existing modules have been significantly upgraded: sample_inelast and eval_inelast now sup­port spin-dependent scattering (see Section 3.4), the monochromator module can simulate a wider range of motions including Doppler drives and phase-space transformers (Section 3.6), and polarizer_sm has been refactored for improved spin handling. Beyond physics capabilities, *VITESS 3.8* enhances graphical usability and maintainability, with a more flexible graphical user interface (GUI) for adding or rearranging instrument components, extended visualization options [including integration with the *GR* framework (Heinen *et al.*, 2020 ▸) and Python-based tools], and systematic automated testing via a Git-based continuous integration system (Section 2.1). The release also introduces an online HTML documentation portal and new tutorial materials, reflecting a broader effort to modernize the codebase and strengthen reproducibility. Together, these developments reinforce *VITESS*’s role as a robust and extensible simulation environment for neutron instrument design, bridging traditional ray tracing with emerging data-driven and AI-assisted modeling approaches.

The project *VITESS* is now hosted in a GitLab repository (https://iffgit.fz-juelich.de/vitess), which facilitates transparent development, community contributions and reproducible builds (see Section 2.1). In what follows, we give a brief introduction to how *VITESS* works and explain some upgrades in terms of software design in Section 2. Then, we present the most relevant, fully deployed, new features that are now available in version 3.8 in Section 3. Finally, we discuss the role of *VITESS* in instrument design for large-scale facilities and the current landscape of Monte Carlo simulation software in Section 4.

## 2. Software design

*VITESS* can be operated through a GUI that facilitates the setup and execution of neutron instrument simulations, a Python interface, or the command line. The software comprises of a set of modular components called ‘modules’ that are linked in a sequential processing chain, known as a ‘pipe’. Each module represents a distinct instrument component (such as a source, guide, chopper or detector) and passes simulated neutron data directly to the next module in the sequence without storing intermediate results. Each module is an independently compiled binary, *i.e.* they are independent programs that communicate with each other by transferring data of the incoming and/or outgoing neutrons. The simulation typically begins with a source module (or an event file) and ends with a detector and a related output module, which visualizes or analyzes the distribution of the detected neutrons.

Intermediate diagnostics can be inserted using monitor or writeout modules without interrupting the data flow. Each module works in a local coordinate system. Coordinate transformations from one module to the following are added up for the instrument and trajectory visualization and to deliver information about the position of all components in a fixed coordinate system. This allows complex instruments to be composed from locally defined reference frames and to be checked to maintain spatial consistency throughout the simulation. The information about the position of all components of the instrument in a fixed coordinate system with its origin at the center of the source is written to an output file in each run of a simulation, allowing the correct position of each component to be checked.

Users can easily define, edit and verify module parameters within the GUI, save configurations for later use, or export them as batch scripts (in shell, Tcl, Perl or Python) for automated execution. This modular and scriptable workflow makes *VITESS* adaptable for both interactive instrument design and reproducible large-scale simulation studies.

To facilitate user contributions, the kernel was tidied up: The structure is very similar in each module, using the same names for corresponding functions. All global parameters are clearly defined in each module and related to the input parameters from the GUI.

### 2.1. Tests and software development environment

The software development environment of *VITESS* has been greatly improved between 2023 and 2025. Developing, testing and releasing of new versions is now fully integrated in a Git system, which is hosted on GitLab as mentioned before. This not only provides access to the open-source code but also facilitates user contributions and enables efficient communication with the maintainers through integrated issue tracking, allowing for user requests. For user contributions, it is possible to open a pull-request on the GitLab platform and follow the contribution guidelines of the repository. To date, users have written about 20% of the *VITESS* modules from scratch and added new features to another 20% of the modules. Corrections are usually carried out by the users, while maintenance work is done by the *VITESS* team.

Any change pushed to the repository triggers an automated compilation (for Windows, MacOS and various Linux distributions) and a testing pipeline. These changes are only accepted if all tests are passed, *i.e.* if the written events agree exactly with reference output files.

Testing the code by running the instrument simulations in the EXAMPLES folder of the repository and comparing them with the results of previous simulations has been standard practice for many years. While most modules are included in at least one of the example instruments, it was not guaranteed that all major options of all modules were tested. Furthermore, getting a wrong result (or no result) does not show directly where the bug is. Therefore, more than 200 module tests were written, where an event file is read, the neutrons are treated by one module in one of its main options and the new neutron parameters are written to an output file. In the complete test, all these output files are compared with reference files. The test will only pass if all output files are identical to the reference files. This means that the comparison happens at the level of individual neutron rays, not on statistical properties over all neutrons. The original condition was that the two files have to be identical; this has been changed to a comparison of values with very high precision to allow for rounding errors, caused by platform-dependent differences in floating-point calculations. This procedure ensures that the development branch is always tested for correctness and can be used for a release at any time, allowing more frequent releases. Even more importantly, a new feature requested by any user can be implemented, tested and merged into the ‘develop’ branch without interfering with other developments. Then, this user can download a compiled development version of *VITESS*, which contains the requested change.

### 2.2. Control commands

**Figure 1:**
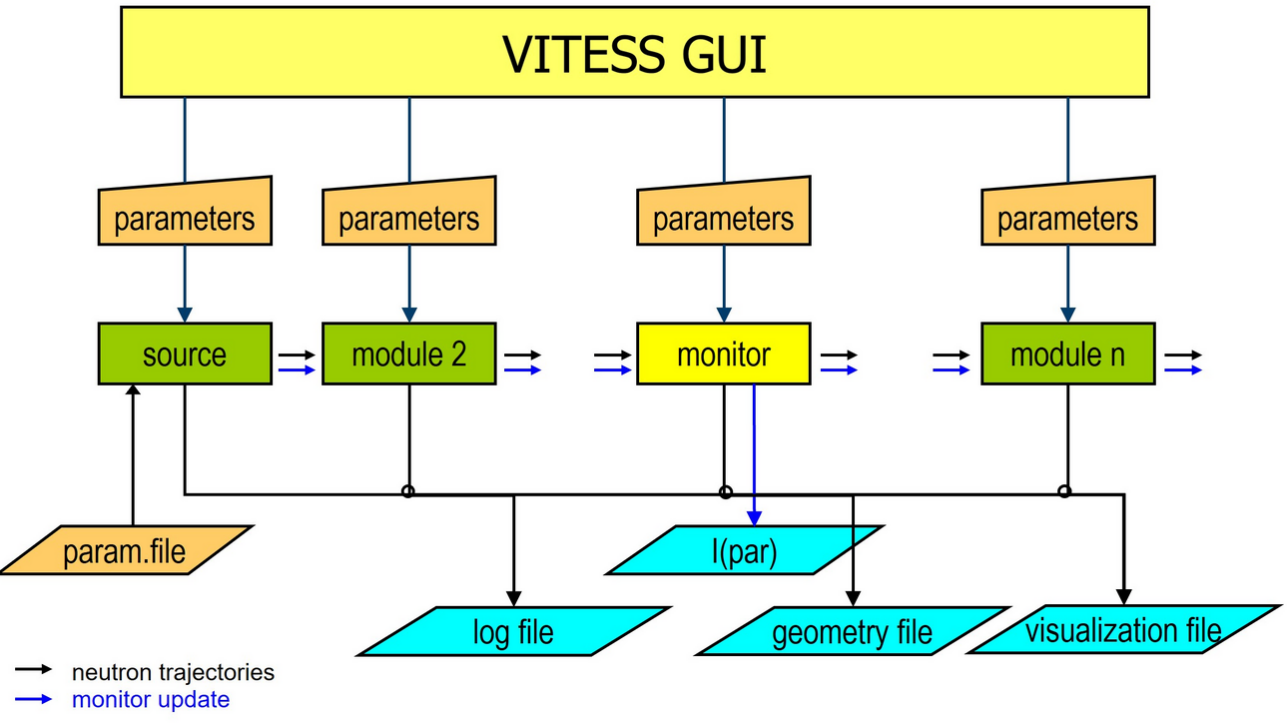
Concept of *VITESS*, showing the stream of events from one module to another including a control code for the monitor updates.

The transfer of neutron data from one module to the following is realized by using the input stream for reading and the output stream for writing. This stream is now also used for communication between the source and any module. An event that is passed from one module to the next can now also contain a command for a monitor, to update the monitor output (see Fig. 1 ▸), which provides the user with intermediate results of long-running simulations.

The monitor update is realized by splitting the total number *N* of neutron ray trajectories into 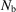 bunches of 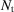 trajectories per bunch, *i.e.*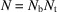. After each bunch of 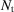 trajectories, the source module creates the event ‘monitor update’ and writes it to the output stream like the neutron data sets. This causes all monitor and evaluate modules to update the output file, which contains intensity or polarization values considering all trajectories processed so far. The output values written after 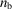 bunches are multiplied by the factor 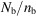 in order that the output data always have a correct normalization. In this way, the numbers do not grow with increasing number of bunches, but the statistics get better. The information about the total number of bunches is stored by the source module in the file simulation.inf, which is generated when the simulation starts and can be accessed by any module. The monitor update during the live run mode does not significantly slow down the simulation if the number of rays within one bunch is chosen to be large enough (one million or higher). Each bunch is fully executed before the update command takes effect. So the increase in time is the ratio of the time for storing the monitor files to the execution time of one bunch, which strongly depends on the instrument and the number of monitors. As an example, in a simulation of an ESS diffractometer with many monitors, we did not see an increase in simulation time going from 100 million rays to 100 times 1 million rays.

## 3. New features

In this section we provide detailed information on the new features that have been included in versions 3.7 and 3.8, which consist of needed upgrades to some modules but also completely new modules. Many of them have been motivated by user requests, and others are intended to provide new functionalities to Monte Carlo simulations.

### 3.1. Reading and writing events

The modules read_in and writeout are used to read and write data of neutron trajectories. Originally, reading and writing were directly done by the first and last module in the pipe, respectively, in *VITESS*’s internal binary data format. The goal was to enable splitting of the instrument simulation into two or more parts. Using the dedicated modules read_in and writeout extended the possible applications drastically. Files can be written in human-readable format (as ASCII files) to check the neutron parameters at any point of the instrument. Simulations can be combined with simulations using other programs, *e.g.* simulations of the target–moderator–reflector (TMR) unit or the detector using *MCNP* (Forster & Godfrey, 2006 ▸), *PHITS* (Niita *et al.*, 2006 ▸) or *Geant4* (Agostinelli *et al.*, 2003 ▸). (This is complemented by the option to write out events of neutron absorption along the guide as a basis for simulations of the guide shielding.)

To enable all possible applications, more formats were included in *VITESS*. Apart from its own data formats, *VITESS* can read and write *McStas* (Willendrup & Lefmann, 2020 ▸) (ASCII and binary), Monte Carlo Particle Lists (MCPL; Kittelmann *et al.*, 2017 ▸), MCNP6 and SSW (used in *MCNP*) files. However, the *VITESS* format is still used in the program as the default format for input and output because it contains additional software-specific parameters, which are needed for some important features of the *VITESS* program: it contains a parameter that distinguishes between rays and control commands (see Section 2.2), an ID parameter that is necessary to trace an individual ray through the instrument, and a color parameter that enables users to mark a ray. Additionally, using its own format allows writing of any kind of subset in any format into any file (*e.g.* to check the positions of the rays), while tools like the MCPL package only allow reading and writing of complete events in binary format.

### 3.2. *NCrystal* integration

The sample_ncrystal module introduces a major extension of the *VITESS* framework by integrating the *NCrystal* (Cai & Kittelmann, 2020 ▸) library for realistic simulations of neutron scattering in crystalline and polycrystalline materials. This integration allows users to model coherent and incoherent scattering, absorption, and spin-dependent processes using material-specific cross sections and scattering kernels provided by *NCrystal*.

Within *VITESS*, neutrons enter the sample volume, where *NCrystal* is queried for the total macroscopic cross section of the configured material. This information is used to account for attenuation and to sample whether and where an interaction occurs along the neutron path. If an interaction takes place, *NCrystal* selects the relevant interaction channel and computes the final neutron state, including energy transfer, scattering angle and spin change where applicable. The updated neutron parameters are then passed back to *VITESS* for further transport through the instrument.

The scattering process follows this sequence:

(1) *VITESS* propagates the neutron through the sample while accounting for attenuation using the total cross section provided by *NCrystal*.

(2) If an interaction occurs, *NCrystal* samples the interaction type and determines the scattering kinematics.

(3) *VITESS* updates the neutron trajectory, including wavelength, direction, position and spin state.

For inelastic scattering events, the energy ℏω and momentum transfer **q** are sampled from *NCrystal’s* normalized inelastic scattering kernel *S*(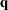, ω), which defines the conditional distribution of final states once the inelastic channel has been selected. Elastic and diffraction contributions are treated separately according to the corresponding cross-section components.

Materials are initialized from *NCrystal* configuration strings, enabling reproducible setups based on existing .ncmat files. Temperature, mosaicity, *d*-spacing cutoffs and other crystal parameters can be specified via the graphical interface. Various sample geometries are supported, and crystal axes can be oriented independently of the sample geometry using configurable offset angles.

The sample_ncrystal module thus provides a comprehensive and physically consistent interface between *VITESS* and the *NCrystal* material database, enabling accurate simulation of neutron–matter interactions while preserving full compatibility with the *VITESS* transport and instrument modeling framework.

### 3.3. The reflectometer sample for digital twins

The original approach of neutron instrument simulation software was to consider only neutrons that were on the way to the sample and detector. No other particles and no nuclear reactions were treated, and if a neutron did not hit the entry of any component it was removed from the simulation. This concept made instrument simulations several orders of magnitude faster than simulations considering nuclear reactions using programs like *MCNP*. That enabled comparing the flux and beam profile at the sample position and diffraction patterns for a variety of instrument setups or even numerical parameter optimization.

However, two new developments require changes of this concept: The first is the interest in background estimation, so that not only the signal but also the signal-to-noise ratio can be estimated. The second is the development of digital twins, which enable users to get experience in handling the instrument and test different instrument settings for their measurement, while instrument responsibles can use them to develop data evaluation software during the instrument construction.

**Figure 2:**
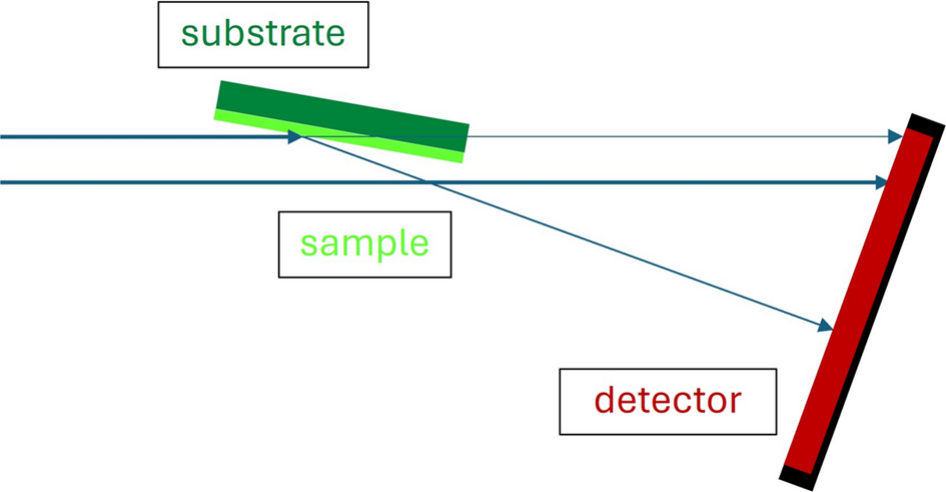
Sketch of the trajectories treated in the *VITESS* module sample_reflectom.

In both cases, removing neutrons not hitting the sample is the wrong strategy and neutrons transmitted from the sample have to be treated as well. These changes have to be included in all sample modules. The reflectometer sample is the first of the original sample modules where this has been realized: treating neutrons not hitting the sample can be switched on or off, while transmitted neutrons are always treated. Additionally, a support of the sample is included, where the transmitted neutron beam is attenuated (see Fig. 2 ▸). This allows a much more realistic simulation of the sample and is a first step towards estimation of background caused by the sample.

Additionally, the features of the module sample_reflectom to add incoherent scattering and to simulate off-specular scattering have been corrected and tested properly.

### 3.4. Spin-dependent inelastic scattering

*VITESS* provides two complementary modules for inelastic neutron scattering simulations. The sample_inelast module models the physical scattering process inside an inelastic sample, including sample geometry, attenuation and the generation of scattered neutrons according to prescribed scattering kernels 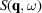.

In contrast, the eval_inelast module evaluates simulated neutron data at the detection stage. It emulates the analysis workflow of inelastic neutron scattering experiments by integrating detected neutron probabilities to obtain intensities corresponding to specific 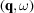 points, without altering neutron trajectories or energies

The two modules thus address different stages of the simulation workflow: sample_inelast describes neutron–matter interactions, while eval_inelast processes the resulting neutron distributions to extract observables. In *VITESS 3.8*, both modules were extended to support spin tracking during inelastic scattering, enabling polarization-dependent simulations beyond purely scalar treatments. This extension applies to nuclear scattering processes and allows neutron spin states to be propagated consistently through inelastic interactions.

The scattering kernel is expressed as 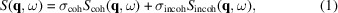
where 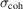 and 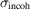 are the coherent and incoherent microscopic scattering cross sections. The functions 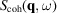 and 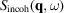 are dimensionless and normalized such that their integral over the considered energy-transfer range equals unity. Under the assumption that multiple scattering is neglected, the simulated scattering intensity remains proportional to the corresponding physical cross sections. The coherent term 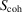 describes energy and momentum transfer according to a parameterized dispersion relation, 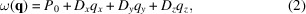
where 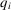 are the components of the momentum transfer and 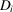 are user-defined dispersion coefficients. This formulation provides a local linear approximation to more complex dispersion relations. Incoherent inelastic scattering is treated using a simplified model in which energy transfer is sampled from a uniform distribution within a user-defined range and scattering directions are assumed isotropic. This approximation is intended to represent generic incoherent background contributions and does not model detailed phonon or magnetic excitation spectra. Spin dependence is introduced for nuclear incoherent scattering by assigning spin-flip events with probability 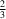, while 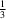 of events preserve the incoming spin: 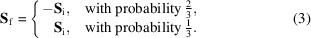
This probabilistic model reproduces the depolarization effect arising from random nuclear spin orientations. Coherent nuclear scattering preserves the neutron spin. Magnetic scattering processes with spin-dependent cross sections are not included in the present implementation. After scattering, the neutron’s updated spin state, energy 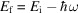 and direction are propagated to subsequent modules. The corresponding eval_inelast routines were updated to handle spin-resolved event data, enabling polarization-dependent analysis of simulated measurements. These extensions provide a consistent framework for polarization-resolved virtual experiments involving nuclear inelastic scattering, while clearly defining the physical scope and approximations of the implemented models.

### 3.5. Artificial-intelligence-based sources

Given recent advances on probabilistic generative modeling, we have included a source module that allows creation of neutron rays by sampling generative models. These models need to be previously trained in the *PyTorch* framework of Python (Paszke *et al.*, 2019 ▸). The models learn the underlying multivariate distributions of MCPL files [as described by Kittelmann *et al.* (2017 ▸)] and provide sampling functionalities.

To use the module source_AI, the basic workflow is as follows. The first requirement is an MCPL file containing samples from which the underlying distribution is learned, to use as source in a *VITESS* simulation. Then, a generative model [provided by Robledo *et al.* (2025 ▸) or self-designed] is trained on the data from the MCPL file in the *PyTorch* framework. The quality of the learned distribution can be assessed using various statistical metrics. In particular, we recommend the use of the maximum mean discrepancy (MMD) test (Gretton *et al.*, 2012 ▸). MMD quantifies the difference between two samples, yielding smaller values when both are likely to be drawn from the same distribution and larger values otherwise. This enables direct comparison between samples generated by the AI model and those obtained from the MCPL file. For reference, the MMD computed between two independent sub-samples from the MCPL file serves as a baseline, since both originate from the same underlying distribution and thus represent an expected ‘good’ MMD value. More information on the selection of the generative model, as well as the training and the assessment of quality, is provided by Robledo *et al.* (2025 ▸). Once the model is trained, then it can be just-in-time (JIT) compiled and exported using the C++ *PyTorch* API (*PyTorch*, 2025 ▸). The JIT-compiled model is the file that should be used in the source_AI module input file box.

The training phase is carried out in *PyTorch* and may represent a significant barrier for users who are not proficient in Python or who lack a background in artificial intelligence. During this phase, the dataset is fed into the selected network architecture, and the model parameters are optimized so as to minimize the negative log-likelihood (equivalent to minimizing the Kullback–Leibler divergence) between the generated distribution and the original dataset. The data are provided to the generative model in mini-batches, and an optimization algorithm such as stochastic gradient descent or Adam is employed. Further details are given by Robledo *et al.* (2025 ▸).

**Figure 3:**
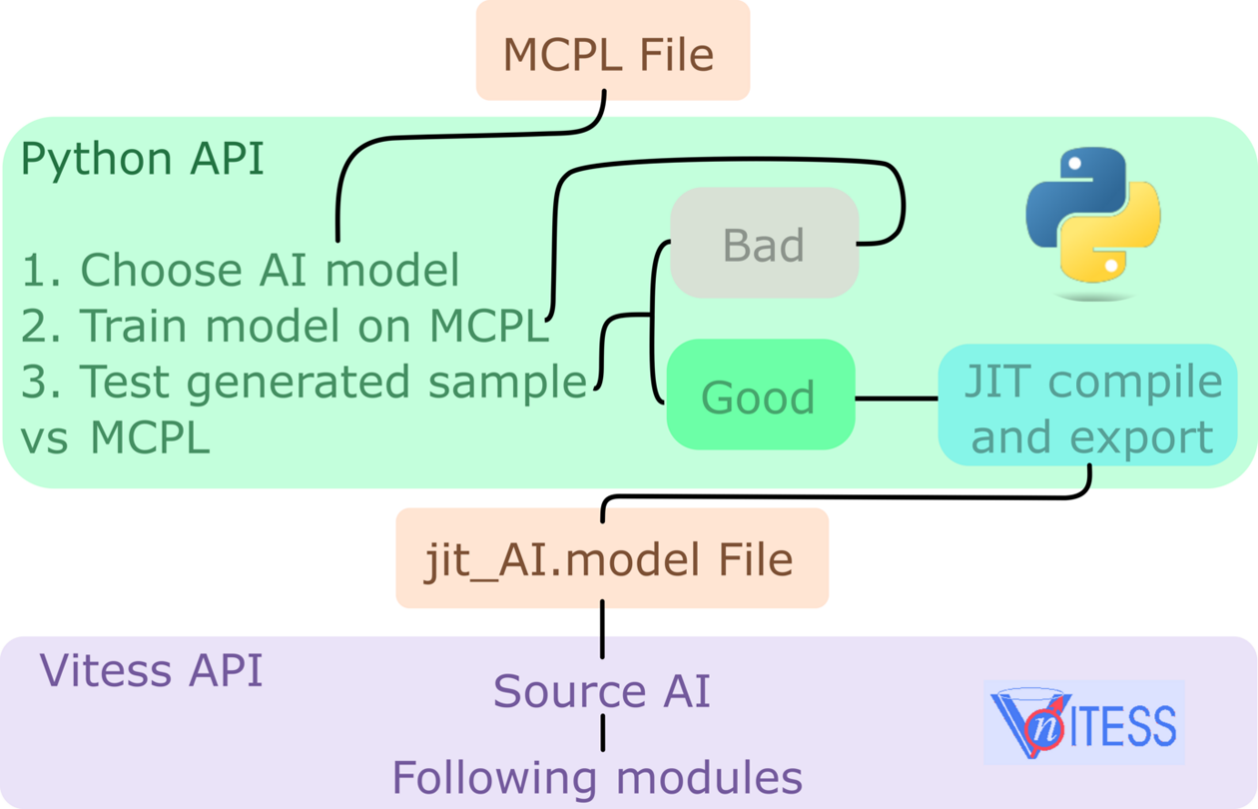
Workflow for using the source_AI module. Models must be trained in Python and need to be JIT compiled to be used by the *VITESS* module.

A schematic of the workflow is shown in Fig. 3 ▸. An MCPL file is provided to the Python API, an AI model is chosen, and then the model is trained on the data in the MCPL file via *PyTorch* and used to sample new neutrons. These neutrons are tested against the original MCPL file until a stopping criterion of the goodness of the generated sample is fulfilled. Afterwards, the model is JIT compiled and exported to a model file, which is then fed to the source_AI module to generate neutrons and pass them on to the next modules.

**Figure 4:**
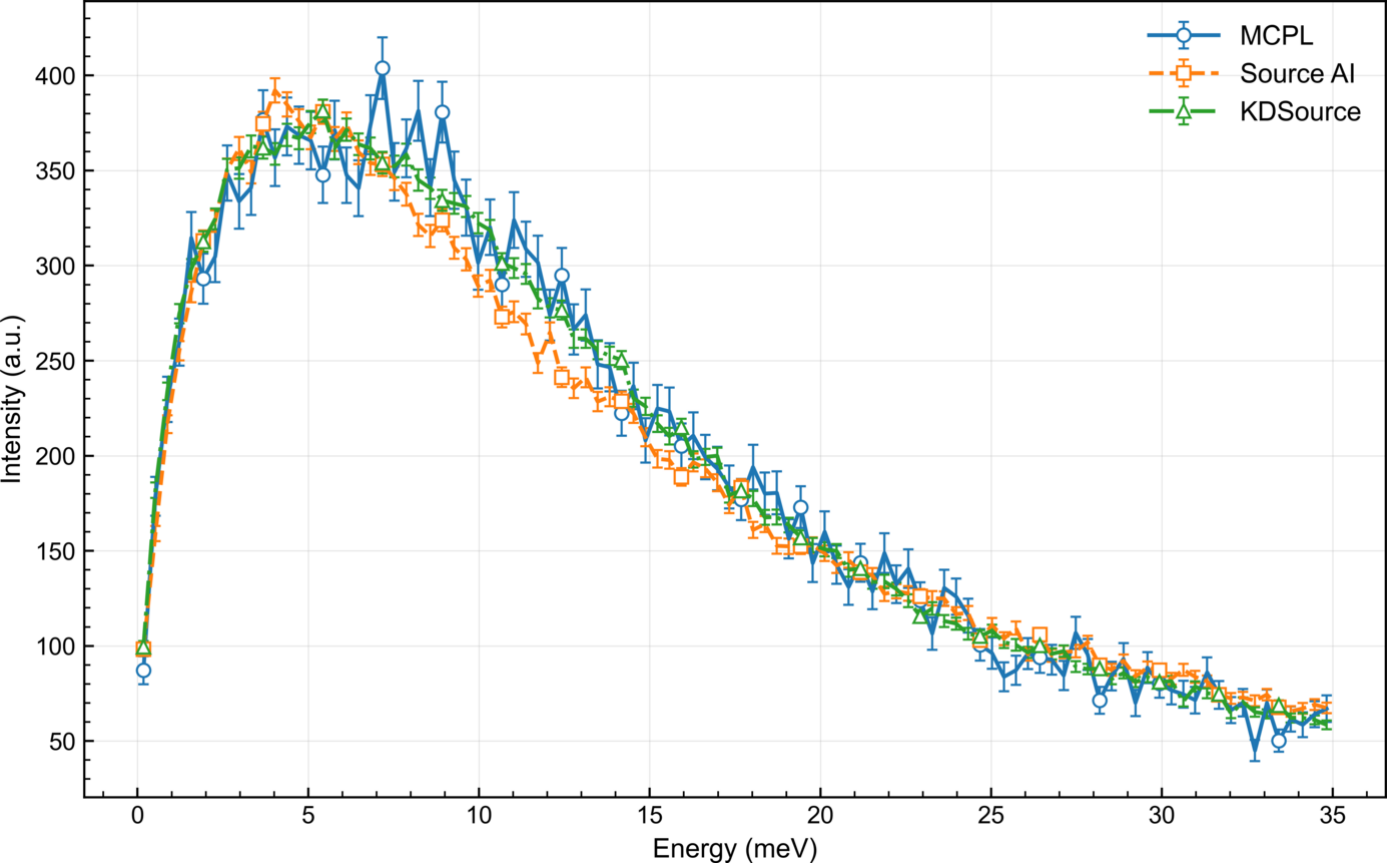
Comparison between different sampling schemes: reading in the MCPL file, sampling with a trained AI model such as a normalizing flow, or sampling with *KDSource*.

An example of the source_AI module in action is shown in Fig. 4 ▸. A normalizing flow with coupling layers (Papamakarios *et al.*, 2021 ▸) is trained on the data of an MCPL file obtained from a *PHITS* simulation at the entrance of a neutron guide. Then *VITESS* is used to sample this model through the source_AI module to further propagate new neutrons inside a beamline. For the moment, available generative models are normalizing flows (Papamakarios *et al.*, 2021 ▸), variational autoencoders (Kingma & Welling, 2014 ▸) and diffusion models (Hoogeboom *et al.*, 2023 ▸). New models can be included as feature requests in the issue tracker of the *VITESS* GitLab repository.

In another development towards AI sources, sampling through *KDSource* (Schmidt *et al.*, 2022 ▸), a tool for Monte Carlo particle source generation, is now possible in *VITESS*. The software *KDSource* employs a method based on multivariate kernel density estimation of the input particle list, enabling a non-parametric reconstruction of the underlying source density. This allows users to sample dynamically (‘on the fly’) directly inside *VITESS*. The main advantage of this approach is that it captures complex, multidimensional correlations in the data without assuming any specific parametric form for the distribution, thereby providing a more accurate and flexible representation of the source characteristics compared with traditional analytic or histogram-based methods.

This upgrade serves the same purpose as the source_AI module in the sense that it generates new neutrons that belong to the distribution of the original file, but it does it in a different manner by perturbing the original particles in the phase-space variable space. Therefore, it does not learn a distribution but rather samples strategically around the known samples. The advantage of this method is that it is faster for sampling and performs well if the number of neutrons in the original particle list is small.

As with the source_AI module, the *KDSource* workflow includes a training stage that must be carried out using its Python API (see the package documentation for details). This process produces an XML source file that stores the optimal kernel bandwidths identified during the kernel density estimation training. To use *KDSource* within a simulation, this source file as well as the original data must be supplied. The data are then randomly sampled and randomly perturbed according to the bandwidths learned during the training phase.

A comparison of the energy distributions for the different sampling schemes explained here can be seen in Fig. 4 ▸, where consistency and agreement is observed between the two new alternatives. The curve labeled ‘MCPL’ shows the result for a simulation using the original dataset as input. Both alternatives presented here provide better uncertainty estimation because they can be oversampled, *i.e.* more neutrons can be generated than the ones in the MCPL file. In this case, the sample size of the models was 10 times larger than that of the MCPL file.

Importantly, the ability of both *KDSource* and source_AI to reproduce the original distribution depends on the quality of their training. In the present case, deviations in the spectral shape indicate that the AI model would benefit from additional training. Nonetheless, the model has successfully learned the underlying distribution and is now fully independent of the original dataset. This makes it lightweight and easily shareable. In contrast, the *KDSource* approach requires access to the original dataset at all times, as it generates new samples by applying perturbations to the empirical data.

Although we illustrate the results only for the energy spectrum, both methods estimate the full multivariate neutron phase space, including positions 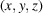, direction vector components 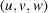 and wavelength λ. The particle weight *p* is only estimated in the source_AI approach, as this feature is not yet supported in the *KDSource* package.

As a final comment, the module introduced in *VITESS 3.8* enables sampling from AI models that is particularly advantageous when iterative tasks must be performed for the same source, for example when running multiple virtual experiments for a given beamline or when exploring different beamline configurations while keeping the source unchanged. Consequently, the novel capability introduced in this section is of potential interest to large-scale facilities seeking to optimize or design instruments. A natural anticipated use case is for neutron facilities that already perform Monte Carlo simulations of their sources to train AI models that reproduce the source distributions at their beam ports. These trained models could then be distributed to users, allowing them to bypass both the need to learn the training procedure and the computationally intensive Monte Carlo simulations required to generate the training datasets.

### 3.6. Moving monochromator

To cover all possible usages of monochromators, the monochromator module is amongst the most complex modules of *VITESS*. The concept is to describe a single-crystal element by parameters like size, orientation, mosaicity, peak reflection *etc*. and then define the geometry of these (identical) crystals separately. This can be done either by defining it through a mathematical function (*e.g.* a vertical cylinder) or by defining the position and orientation of each element in a table. (This table is also generated if a mathematical function is chosen and can then be edited to match the real arrangement exactly.)

In spite of this flexibility, there are also monochromator arrangements that could not be realized with this module, either because the monochromator is moving or because it consists of more than one layer. The phase-space transformer (PST) used in backscattering instruments like SPHERES at MLZ (Wuttke *et al.*, 2012 ▸) or IN16B at ILL (Appel *et al.*, 2018 ▸) is one example. Here, the monochromator is mounted on a rotating disc chopper and, in the case of SPHERES, consists of three layers. In the same instruments, monochromators are mounted on a Doppler drive to create a periodic variation of the wavelength.

Therefore, two new features were implemented to enable simulations of these instrument. One is an extension of the crystal element positions to allow also different layers, *i.e.* variations of the positions in all three dimensions, for both calculated geometries and geometries from tables.

The other new feature is the possibility to treat a moving monochromator. Three kinds of movements are enabled: oscillation along the beam axis (for the Doppler drive), rotations about a horizontal axis at a distance from the rotation axis (for the PST) and rotation about a vertical axis through the origin of the coordinate system. In all cases, the neutron is first propagated to the point of reflection, and then the position, velocity and spin of the neutron are transformed to the coordinate system of the moving monochromator before the reflection is performed. Finally, the neutron parameters are transformed back to a fixed coordinate system, in this case to the output frame, and the neutron is propagated to the exit of the component.

### 3.7. Larmor precession in the He-3 polarizer

The module polarizer_he3 in *VITESS* has been improved to handle the full Larmor precession of neutron spins as they traverse the polarizing gas cell and guide fields. For each neutron, the code calculates the local spin orientation relative to the magnetic field and propagates the spin vector through multiple domains, accounting for time of flight, field strength and trajectory geometry. Absorption probabilities are spin dependent, with the chance of neutron absorption determined by the projection of the spin along the ^3^He polarization axis. The module integrates polarization and transmission data either from analytical calculations or from external files, allowing accurate estimation of the outgoing neutron intensity and polarization. At each step, the neutron spin is rotated using a Larmor rotation matrix corresponding to the accumulated phase shift, and deviations from parallel or antiparallel alignment are flagged for diagnostic purposes. This upgrade ensures that simulations now correctly reflect the interplay between neutron spin precession and absorption in complex magnetic field configurations, enhancing the fidelity of polarized beam virtual experiments.

### 3.8. Neutron refraction on a prism

A new module, prism, has been introduced in *VITESS 3.8* to simulate neutron refraction and attenuation in single prisms or arrays of identical prisms. The module allows detailed geometric and material parameterization, including prism base width and height, array configuration (rows and columns), and material properties such as scattering length density, absorption and incoherent cross sections. Each prism is modeled as a triangular element within a 3D matrix, enabling realistic simulation of complex optical assemblies such as gravitational correction arrays or wavelength-separating devices. The refraction follows the wavelength-dependent refractive index 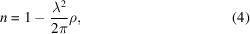
where 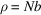 is the scattering length density, *N* the number density of scatterers and *b* the coherent scattering length. It provides accurate angular dispersion for long-wavelength neutrons. The module prism extends the suite of modules for the simulation of neutron optical elements, consisting so far of two modules for mirrors (plane and elliptical) and one for a lens. Its functionality supports beam deviation and includes optional absorption effects for non-refracted trajectories. The module thus enables realistic studies of beam deflection, gravity compensation and chromatic dispersion in neutron optical systems.

## 4. Discussion and future perspectives

*VITESS* is a Monte Carlo simulation package designed for the development of neutron instruments and, with the novel features presented in this work, for supporting the validation and interpretation of experimental measurements. In general, cross-validation with experimental data is recommended whenever such data are available and applicable. However, for studies focused on instrument design or hypothetical configurations, direct comparison with measurements may not be possible. In such cases, simulations with software like *VITESS*, *McStas* (Willendrup & Lefmann, 2020 ▸) or *McVine* (Lin *et al.*, 2016 ▸) provide necessary and well-established tools.

When compared with other Monte Carlo neutron simulation packages, *VITESS* occupies a distinctive position in terms of philosophy, architecture and typical use cases. Like *McStas*, *VITESS* employs a modular structure that allows users to construct virtual instruments by linking predefined components, yet its ray-tracing paradigm is implemented through independently executable modules that communicate via data files, providing a high degree of flexibility and interoperability. It allows creating new neutron trajectories during the simulation, *e.g.* by scattering a neutron on all Bragg lattice planes, which makes the simulation in some cases much faster compared with other packages, especially for single-crystal diffraction.

*McStas*, by contrast, uses a compiled script-based approach, where instrument descriptions are defined in a domain-specific language and compiled into C code, often resulting in higher computational efficiency for large simulations. Meanwhile, *McVine* adopts a hierarchical object-oriented framework built in Python and C++, emphasizing detailed scattering kernels and enabling close integration with data reduction and analysis workflows, particularly for inelastic neutron scattering. *VITESS*’s design philosophy prioritizes transparent data flow and modular exchangeability, making it particularly suitable for prototyping, testing new instrument concepts, and coupling with external tools such as MCPL or data analysis pipelines.

Having multiple Monte Carlo neutron tracing software packages is essential for a robust and innovative neutron science community. Each code (whether *VITESS*, *McStas*, *McVine* or others) embodies distinct design philosophies, simulation strategies and areas of specialization that collectively enrich the landscape of neutron instrument modeling. Diversity in tools fosters healthy cross-validation, helping researchers identify numerical biases, benchmark results and increase confidence in simulation outcomes. It also encourages methodological innovation, as different frameworks explore new algorithms, modular architectures or interfaces that push the field forward. The coexistence of multiple independently developed simulation environments ultimately strengthens reliability, flexibility and scientific progress within the neutron community.

There are many on-going developments of *VITESS* that suggest a bright future for the software. In *VITESS 4*, the GUI and storage format of instruments will undergo an important update: Qt will be used for the GUI and YAML files will be used to save the instruments in a structured and well-defined way. As these files are human readable, they can be used to build, combine and change instruments. The approach to use the data stream not only for neutrons (see Section 2.2) will be extended to reset the monitors and change parameters during a simulation, which will provide a digital twin close to a real instrument. Furthermore, the possibility of reading in NeXus (Klosowski *et al.*, 1997 ▸) files with the associated instrument definition files is a feature under development that will allow further processing of *VITESS*-simulated data in common data analysis software like *Mantid* (Arnold *et al.*, 2014 ▸).

A *vitess-python *library (https://iffgit.fz-juelich.de/vitess/vitess-python) is currently under development, and it will enable programmatic instrument definition and integration with Python-based workflows. At the same time, plans for a *VITESS* version based on C++ are being developed, which will improve the performance on GPUs and facilitate the addition of new features or new modules. All this reinforces *VITESS* as a modern, extensible framework for neutron instrument design and virtual experimentation.
